# Inhibition of Reverse-Mode Sodium-Calcium Exchanger Activity and Apoptosis by Levosimendan in Human Cardiomyocyte Progenitor Cell-Derived Cardiomyocytes after Anoxia and Reoxygenation

**DOI:** 10.1371/journal.pone.0085909

**Published:** 2014-02-03

**Authors:** Ping-Chun Li, Ya-Chi Yang, Guang-Yuh Hwang, Lung-Sen Kao, Ching-Yuang Lin

**Affiliations:** 1 Department of Surgery, Division of Cardiovascular Surgery, China Medical University Hospital, Taichung, Taiwan; 2 Department of Life Science, Tunghai University, Taichung, Taiwan; 3 Department of Life sciences and Institute of Genome Sciences, National Yang-Ming University, Taipei, Taiwan; 4 Clinical Immunology Center, China Medical University Hospital, Taichung, Taiwan; 5 College of Medicine, China Medical University, Taichung, Taiwan; University of Pecs Medical School, Hungary

## Abstract

Levosimendan, a known calcium sensitizer with positive inotropic and vasodilating properties, might also be cardioprotective during ischemia-reperfusion (I/R) insult. Its effects on calcium homeostasis and apoptosis in I/R injury remain unclear. Na^+^/Ca^2+^ exchanger (NCX) is a critical mediator of calcium homeostasis in cardiomyocytes, with reverse-mode NCX activity responsible for intracellular calcium overload and apoptosis of cardiomyocytes during I/R. We probed effects and underlying mechanisms of levosimendan on apoptosis and NCX activity in cultured human cardiomyocyte progenitor cells (CPC)-derived cardiomyocytes undergoing anoxia-reoxygenation (A/R), simulating I/R *in vivo*. Administration of levosimendan decreased apoptosis of CPC-derived cardiomyocytes induced by A/R. The increase in reverse-mode NCX activity after A/R was curtailed by levosimendan, and NCX1 was translocated away from the cell membrane. Concomitantly, endoplasmic reticulum (ER) stress response induced by A/R was attenuated in CPC-derived cardiomycytes treated with NCX-targeted siRNA or levosimendan, with no synergistic effect between treatments. Results indicated levosimendan inhibited reverse-mode NCX activity to protect CPC-derived cardiomyocytes from A/R-induced ER stress and cell death.

## Introduction

Ischemia-reperfusion (I/R) myocardial injury is a common clinical occurrence: e.g., in aortic de-clamping after cardioplegic arrest in cardiac surgery or prompt therapeutic restoration of blood supply in acute myocardial infarction. Mechanisms mediating reperfusion injury include altered metabolism and Ca^2+^ handling, oxidative stress, microvascular dysfunction, and inflammation [1]. Tremendous effort has been devoted to strategy for ameliorating myocardial reperfusion injury [2], as it carries definite implications in cardiomyocyte survival and myocardial function [3].

Cardinal feature of higher cytosolic Ca^2+^ concentration ([Ca^2+^]_i_) in cells subject to I/R plays a pivotal role in subsequent cellular damage: e.g., hypercontracture as a direct result of excessive [Ca^2+^]_i_. Along with other physiological derangements, excessive [Ca^2+^]_i_ causes arrhythmia, myocardial stunning, and cell death by necrosis or apoptosis. In cardiomyocytes, excitation-contraction coupling is mediated by cyclic changes of [Ca^2+^]_i_, which in turn is regulated by several Ca^2+^ channels. The Na^+^/Ca^2+^ exchanger (NCX) on plasma membrane, 10–15 times more efficient than Ca^2+^-ATPase, is the major channel for Ca^2+^ extrusion during diastole normally [4]. Depending on membrane potential and Na^+^ or Ca^2+^ gradients across plasma membrane, NCX functions in either forward mode (extracellular Na^+^ in exchange for intracellular Ca^2+^) as Ca^2+^ efflux mechanism in physiological conditions, or reverse mode (extracellular Ca^2+^ in exchange for intracellular Na^+^). NCX working in reverse mode is responsible for elevated [Ca^2+^]_i_ that occurs during ischemia and early reperfusion [5], [6], [7]. With cessation of blood flow, ATP is depleted when supply of oxygen and energy source are discontinued abruptly. The Na^+^/K^+^ pump, main Na^+^ extrusion pathway, comes to a halt, leading to accumulation of Na^+^ in cytosol. Higher intracellular Na^+^ concentration in turn reverses direction of action of plasma membrane NCX, causing Ca^2+^ influx. In early reperfusion, as extracellular H^+^ is washed away by re-established blood flow, Na^+^ influx through Na^+^/H^+^ exchanger aggravates Na^+^ and again through the action of reverse mode NCX, Ca^2+^ overload.

Ischemia induces accumulation of misfolded proteins in endoplasmic reticulum (ER) as well as endoplasmic reticulum stress (ERS) response [8]. Expression of glucose-regulated protein 78 (GRP78), an ER chaperone, increases in such response and can serve as a marker of ERS [8]. Intense or persistent ERS can initiate apoptosis, either by cleavage of pro-caspase 12 or ER-mitochondrial crosstalk, with subsequent activation of downstream caspase cascade [8]. Levosimendan, a Ca^2+^-sensitizing inotrope [9], may help preserve cardiomyocytes in setting of I/R [10]–[17]. In animal experiments, levosimendan-conferred protection from I/R-induced apoptosis has been attributed to its activation of mitochondrial ATP-dependent K^+^ channels.[10]–[12] The present study evaluated effects of levosimendan on simulated I-R injury and apoptosis in human cardiomyocyte progenitor cell (CPC)-derived cardiomyocytes *in vitro*, plus involvement of NCX activity and ERS-induced apoptosis pathway in the mechanism. Among isoforms of NCX, ubiquitous NCX1 is highly expressed in the heart [4]. Our data was the first to suggest inhibition of NCX1 activity by levosimendan protecting CPC-derived cardiomyocytes against I/R-induced apoptosis.

## Materials and Methods

### Human Cardiomyocyte Progenitor Cell (CPC)-derived Cardiomyocytes Culture

This research was approved by China Medical University Hospital Institutional Review Board, written informed consent obtained from each patient’s guardian or caretaker. Human cardiomyocyte progenitor cells were isolated from right atrial auricle specimens of individuals undergoing cardiac surgery, which has been a frequently adopted method for isolation of human cardiac progenitor cells [18], [19]. There were 10 patients with equal distribution in both genders, all less than 18 years of age, with various congenital heart diseases: ventricular septal defect, atrial septal defect, endocardial cushion defect and tetralogy of Fallot. Tissue specimens were washed, minced and maintained in growth medium composed of 1∶3 Medium 200 (supplemented with LSGS) and M199 containing 1×MEM nonessential amino acid, 1×penicillin/streptomycin, 10 ng/ml bFGF, and 10% fetal bovine serum. Growing cells were harvested and subcultured on reaching 90% confluence and could be passaged for up to six generations. CPCs were subsequently cultured into CPC-derived cardiomyocytes in differentiation medium (Iscove’s Modified Dulbecco’s Medium containing equal volume of F12, 2 mM L-glutamine, 2% horse serum, 1×MEM nonessential amino acid, 2 ug/ml transferrin, 2 ug/ml insulin, and 1×penicillin/streptomycin). These CPC-derived cells were checked for expression of cardiomyocyte-specific α-sarcomeric actinin [19] and used for experiments. Electrophysiological characteristics of cardiomyocytes, including action potential duration and peak L-Type calcium current, were also measured [20].

### Ischemia-reperfusion Simulation *in vitro*: Anoxia-reoxygenation (A/R)

**Figure 1:**
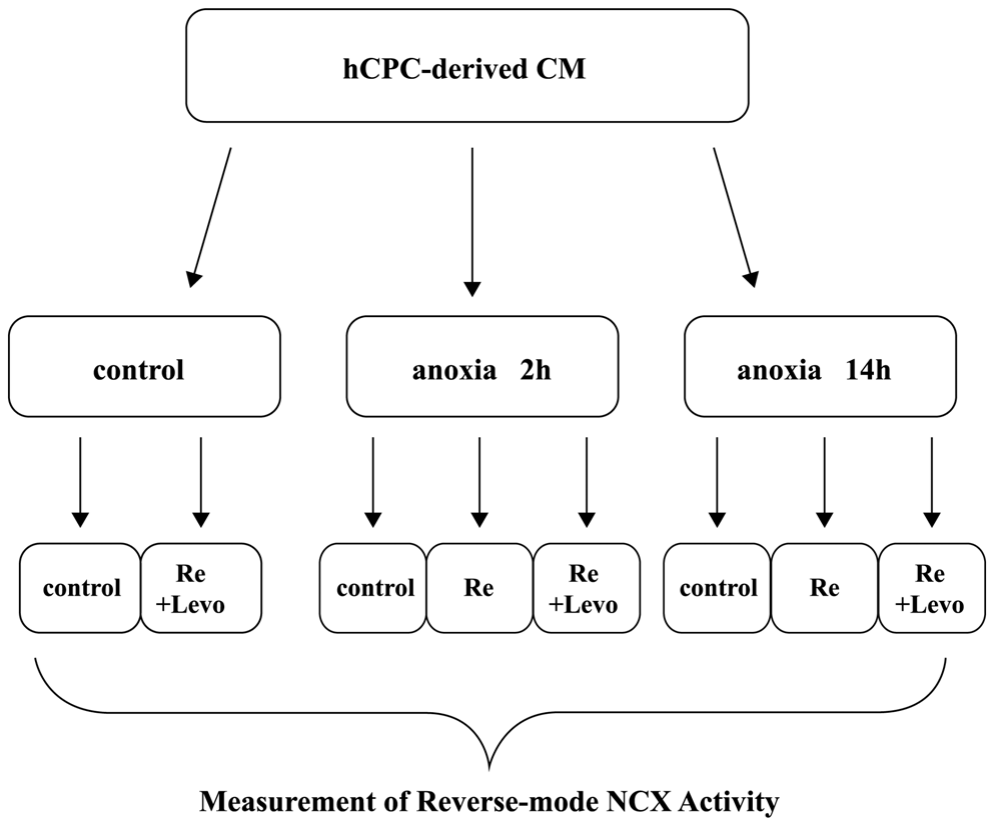
Schematic diagram for experimental procedures of anoxia-reoxygenation. CM: cardiomyocyte; hCPC: human cardiomyocyte progenitor cell; Levo: levosimendan; Re: reoxygenation.

To simulate I/R, CPC-derived cardiomycytes were incubated in a hypoxic chamber (Biosphenix) filled with 95% N_2_ and 5% CO_2_ for 2 or 14 hours in culture medium deprived of glucose and serum, with glucose-free DMEM (Gibco-BRC) supplemented with 100 U/ml penicillin and 100 ug/ml streptomycin. This anoxic period was followed by reoxygenation by transferring cells to a normoxic incubator in 95% air/5% CO_2_ for 2 hours in normal culture medium. Levosimendan 2 uM was added at the beginning of reoxygenation for the experimental group, experimental procedures illustrated in Figure 1.

### Antibodies

The method to generate antibodies against NCX1 was described in our published report [4]. Antibodies against NCX1 were generated, recognizing full-length NCX1 overexpressed in HEK293T cells (with no detectable endogenous NCX) and endogenous mouse cardiac NCX1 [4]. Specificity of antibodies was established by demonstrating that addition of another antigen, peptide sequence of NCX1 intracellular loop, competes out bands formed by NCX1 and antibodies. Alexa Fluor 546 phaloidin and Alexa Fluor 555 were purchased from Molecular Probes (Invitrogen). Donkey anti-mouse IgG-FITC was purchased from Jackson ImmunoResearch Laboratories (West Grove, PA). Anti-GRP78 (1∶2000, Abcam, Cambridge, MA), anti-caspase-12 (1∶2000, Abcam, Cambridge, MA) and anti-β-actin (1∶30000, Sigma, St.Louis, MO) were obtained.

### Measurement of Reverse-Mode NCX1 Activity

Three pieces of 12 mm cover-glass were put in a 3.5 cm dish. Human CPC-derived CMs were cultured on gelatin-coated 12 mm cover-glass (1×10^4^ cells/dish), incubated with 10 uM fura-2 acetoxymethyl ester (AM) at 37°C for 2 h, then loaded with Na^+^ by immersion in Ca^2+^-free Na^+^ loading buffer (145 mM NaCl, 5 mm KCl, 1 mM MgCl_2_, 100 uM ouabain, and 10 mM HEPES, pH 7.4) for 10 min at 37°C. Isolated CPC-derived cardiomyocytes were selected to measure reverse-mode NCX1 activity by abruptly replacing Ca^2+^-free buffer with Na^+^-free buffer (145 mM N-methylglucamine, 5 mM KCl, 1 mM MgCl_2_, 2 mM CaCl_2_, 1 mM ouabain, and 10 mM HEPES, pH 7.4). Fura-2 fluorescence ratio obtained by 340 nm and 380 nm excitation (F_340_/F_380_) was monitored by inverted microscope (IX-70, Olympus Co., Tokyo, Japan) using a 40x oil immersion objective (UAPO 40x oil/340; N/A:1.35; Olympus Corporation, Tokyo, Japan). Amplitude of [Ca^2+^]_i_ rise upon replacement of buffers was then calculated as a measurement of reverse-mode NCX activity [4].

### Fluorescence-activated Cell Sorting (FACS) Analysis

Apoptotic cells were quantified by propidium iodide (PI) and DiOC6 staining with FACS analysis [21]–[23]. Cells plated in 60-mm Petridishes at 8×10^4^ cells/plate in solution containing 50 mg/mL PI and 20 nM DiOC6 (Molecular Probes, Sunnyvale, CA) were analyzed by FACS (FL-3 channel). Ratio of apoptotic cells was detected by DiOC6 fluorescence, results obtained for both experimental and control groups.

### Immunocytostaining and Fluorescence Microscopy

Human CPC-derived CMs grown on cover slips were rinsed three times with PBS and fixed with 3.7% formaldehyde in PBS for 20 min at room temperature. Samples stained with appropriate primary antibodies were incubated with FITC-conjugated goat anti-mouse antiserum at 4°C overnight, cover slips mounted with Vectashield (Vector Laboratories, Burlingame, CA) and viewed under inverted confocal microscope (Zeiss Axiovert 200 M, Carl Zeiss, Jena, Germany) with 63×/1.4 Plan-Apochromat objective.

### Transient Transfection *in vitro*

CPC-derived cardiomyocytes were transiently transfected with NCX siRNA (Invitrogen, Carlsbad, CA) using HiPerFect Transfection Reagent (QIAGEN, GmbH, Hilden, Germany) according to the manufacturer’s instructions. Meanwhile, cells were transfected with scrambled siRNA as control; these were used for experiments after transfection for 48 hours.

### Subcellular Fractionation

Cytoplasmic and membrane fractions of human CPC-derived cardiomycytes were prepared with a CNM compartment protein extraction kit (BioChain, Hayward, CA) according to manufacturer's instructions. Briefly, transfected cells (3×10^6^ cells) were harvested, washed with PBS, and then centrifuged at 500×*g* for 5 min. Cell pellet was homogenized in 200 ul of buffer C plus protease inhibitors, rotated at 4°C for 20 min, then centrifuged at 4°C, 12000×g for 20 min. Cytoplasmic proteins in the supernatant were collected, pellet washed with 400 ul of buffer W and resuspended in 100 ul of buffer N at 4°C for 20 min. Nuclear proteins were extracted and recovered in the supernatant after centrifuge at 4°C, 12000×g for 20 min. Finally, to obtain membrane proteins, the cell pellet containing cell debris was extracted with 100 ul of buffer M and rotated at 4°C for 20 min. Supernatant was centrifuged at 4°C, 12000×g for 20 min. Membrane proteins in supernatant were collected, samples from these fractions denatured and subjected to SDS-PAGE.

### Western Blot Analysis

Total proteins from CPC-derived cardiomyocytes were extracted by RIPA lysis buffer (Millipore, Bilerica, MA), as per manufacturer’s instructions. Proteins (30 ug) were run on a 10% SDS-PAGE gel, transferred onto PVDF membranes, and blocked. Membranes were incubated with primary antibodies: anti-NCX (1∶50000.), anti-GRP94 (1∶5000, GeneTex, San Antonio, TX), anti-GRP78 (1∶2000, Abcam, Cambridge, MA), anti-caspase-12 (1∶2000, Abcam), anti-GAPDH (1∶10000, GeneTex), anti-pan-cadherin (1∶10000, Santa Cruz Biotechnology, Santa Cruz, CA) and anti-β-actin (1∶30000, Sigma, St. Louis, MO) overnight at 4°C. Membranes were extensively washed and incubated with horseradish peroxidase-labeled immunoglobulin G (Jackson ImmunoResearch, West Grove, PA), immunoreactive bands detected by chemiluminescence methods (Millipore) and visualized on X-ray films (Kodak, Rochester, NY). Densitometric analysis was performed by Image J software (NIH, Bethesda, MD) [19].

### Statistics

Statistical analyses used SPSS version 13.0 software (SPSS, Chicago, IL). Data were presented as mean ± standard deviation (S.D.), intergroup comparisons performed with Student's t-test or one-way ANOVA followed by Tukey’s post-hoc test. *p*<0.05 was considered statistically significant.

## Results

### Levosimendan Protects Cultured CPC-derived Cardiomyocytes Against Anoxia-reoxygenation (A/R)-induced Apoptosis

**Figure 2:**
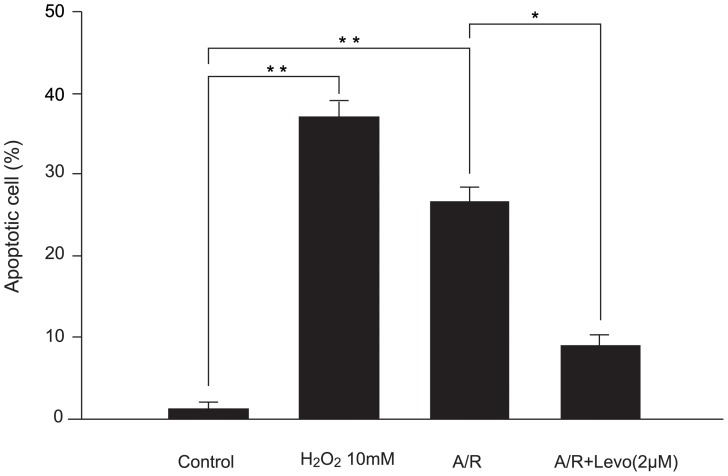
Levosimendan protects against anoxia/reoxygenation (A/R) induced apoptosis in cultured human cardiomyocyte progenitor cell-derived cardiomyocytes. Each point represents mean ± S.D. of six independent experiments. A: anoxia; Levo: levosimendan; Re: reoxygenation (*p<0.05 and **p<0.01).

Effect of levosimendan on apoptosis of CPC-derived cardiomyocytes subjected to A/R was tested, with hydrogen peroxide at 10 mM as positive control. Adding levosimendan at reoxygenation reduced apoptotic CPC-derived cardiomyocytes from 27.6±2.4% to 8.2±2.1% (P<0.05) (Figure 2).

### Increased Reverse Mode NCX Activity After A/R Abolished by Levosimendan

**Figure 3:**
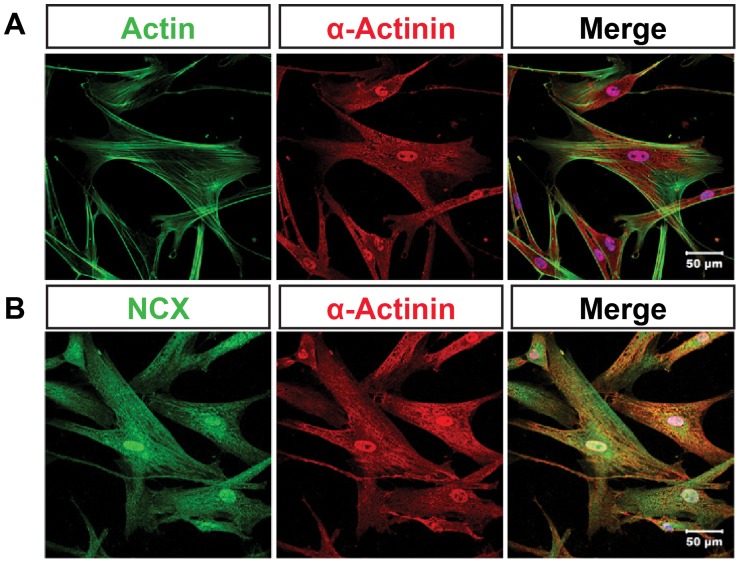
Subcellular localization of NCX in cultured human cardiomyocyte progenitor cell (hCPC)-derived cardiomyocytes. A, Confocal analysis of localization of actin (green) and α-actinin (red) in hCPC-derived cardiomyocytes. B, Confocal analysis of localization of NCX (green) and α-actinin (red) in hCPC-derived cardiomyocytes.

**Figure 4:**
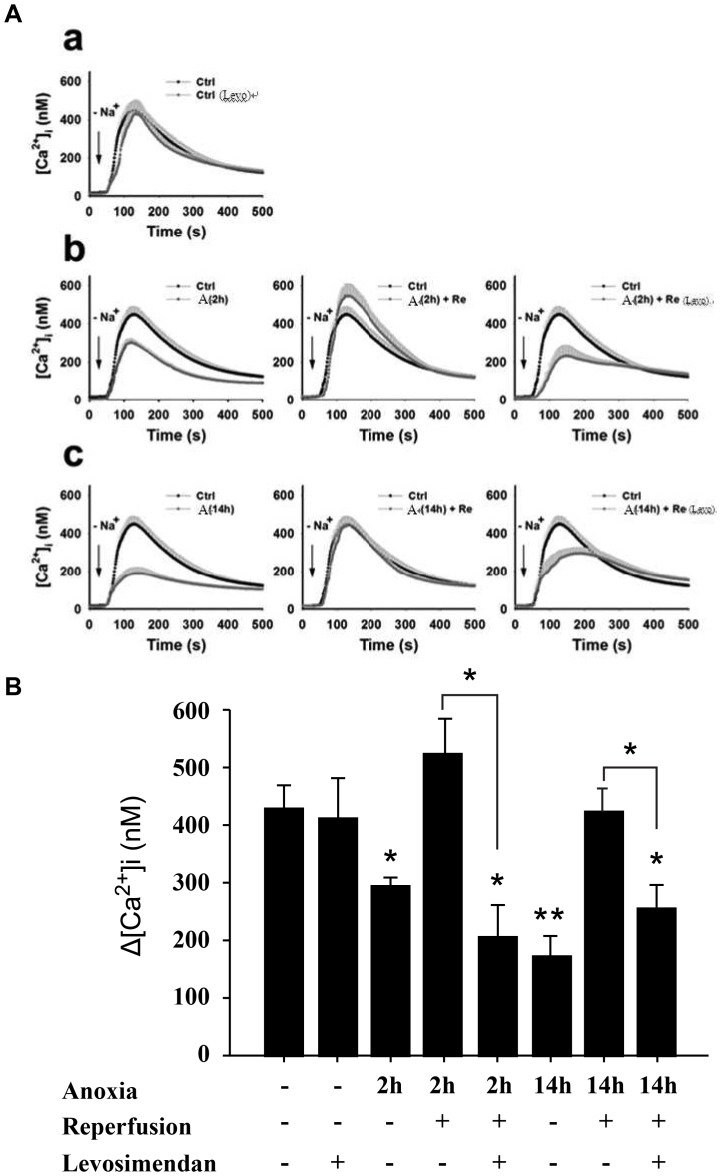
Effect of levosimendan on NCX activity in cultured human cardiomyocyte progenitor cell (hCPC)-derived cardiomyocytes after anoxia/reoxygenation (A/R). (A) Reverse-mode NCX activity was measured with or without treatment of Levosimendan under control conditions (a), after anoxia for 2 hours (b), and after anoxia for 14 hours (c). Reverse-mode NCX activity 2 and 14 hours of anoxia were determined; higher expression of GRP78 and caspase 12 did not reach statistical significance until 14 hours of anoxia (Figure 7). (B) NCX activity was estimated by amplitude of increase in intracellular calcium concentration. (Δ[Ca^2+^]_i_ = peak [Ca^2+^]_i_ - basal [Ca^2+^]_i_). (*n = *15 for each experimental group). Values are expressed as mean ± S.D. from three independent trials. *p<0.05 versus untreated control; **p<0.01 versus untreated control and anoxia for 2 h. Similar results emerged from three independent experiments. A: anoxia; Ctrl: control; Re: reoxygenation; Levo: Levosimendan.

To identify CPC-derived cardiomyocytes, sarcomeric specific α-actinin were detected and cells co-stained with fluorescently labeled phaloidin (Figure 3A). Expression of sodium-calcium exchanger in CPC-derived cardiomyocytes was examined by immunocytochemistry with immunofluorescent microscope (Figure 3B). Under normoxic condition, administering levosimendan does not affect reverse-mode NCX activity in CPC-derived cardiomyocytes (Figures 4Aa and 4B). Reverse-mode NCX activity was decreased after anoxia for 2 h, and activity was reduced even more dramatically after anoxia for 14 h. After reoxygenation for 2 hours, however, there was rebound in NCX activity: much more Ca^2+^ transported into cells than controls (Figure 4Ab). Addition of levosimendan during reoxygenation suppressed heightened post-anoxic NCX activity to below control level (Figures 4Ab, 4Ac, 4B).

### Levosimendan Decreased Plasma Membrane NCX Localization during Reoxygenation

**Figure 5:**
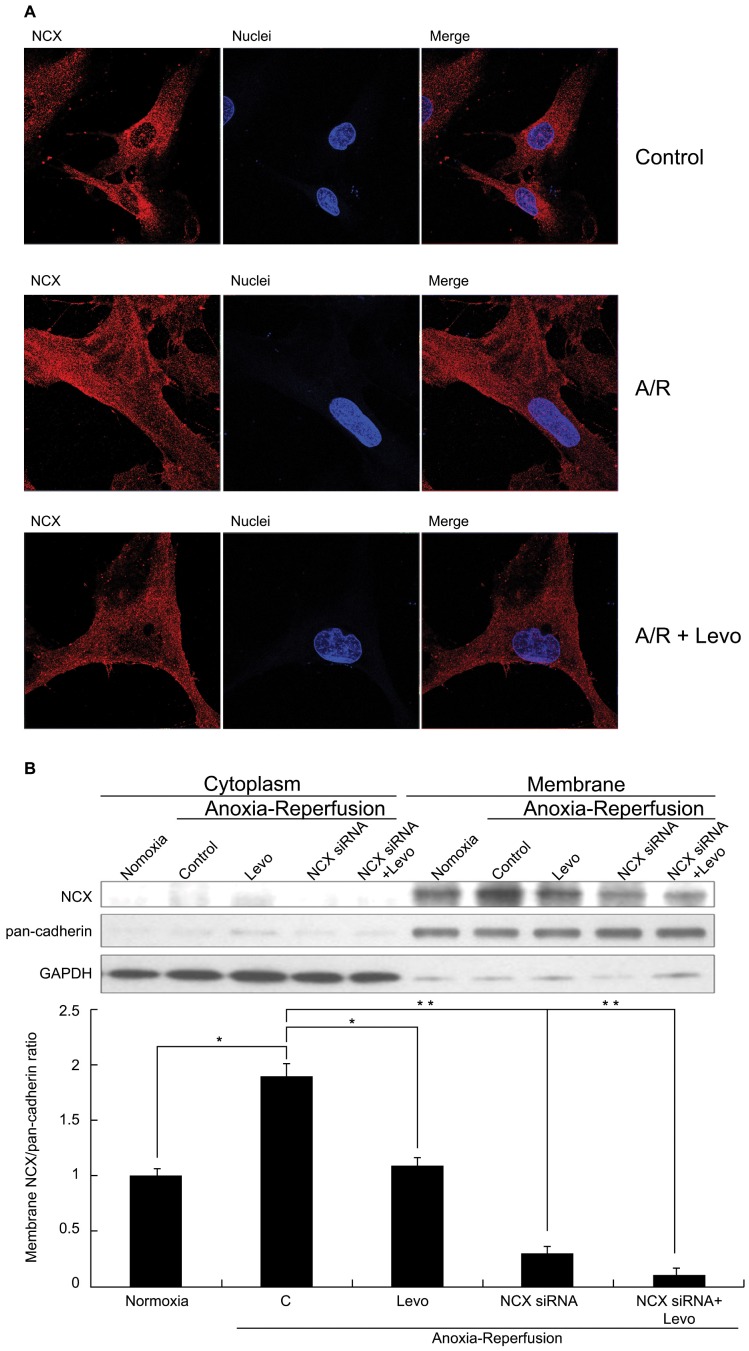
NCX expression on cellular membrane during A/R. (A) Double labeling of cultured human cardiomyocyte progenitor cell(hCPC)-derived cardiomyocytes with NCX (red) and Nuclei (blue). Upper row: untreated control (400X). Middle row: increased NCX expression on cellular membrane and cell swelling after A/R (800X). Lower row: levosimendan administered at re-oxygenation and reduced cellular swelling (800X). (B) Western blot for NCX quantification on cellular membrane. Each point represents mean ± S.D. of three independent trials, *p<0.05 and **p<0.01. A/R: anoxia-reoxygenation; Levo: Levosimendan.

To explain the decrease in NCX activity with levosimendan administration, we examined NCX localization, as NCX can only exert its action of Na^+^-Ca^2+^ exchange when localized to cellular membrane. Such localization of NCX increased after A/R (Figure 5A middle row and Figure 5B) compared to controls (Figure 5A upper row). When levosimendan was administered at reoxygenation, membrane localization of NCX was decreased (Figure 5A lower row and Figure 5B). In addition, cell size apparently increased after A/R, probably via osmosis due to greater intracellular concentration of Na^+^ and other solutes.

### Levosimendan Abrogated GRP78 and GRP94 Expression Enhancement and Caspase 12 Activation in Anoxia-reoxygenation Induced ERS Response

**Figure 6:**
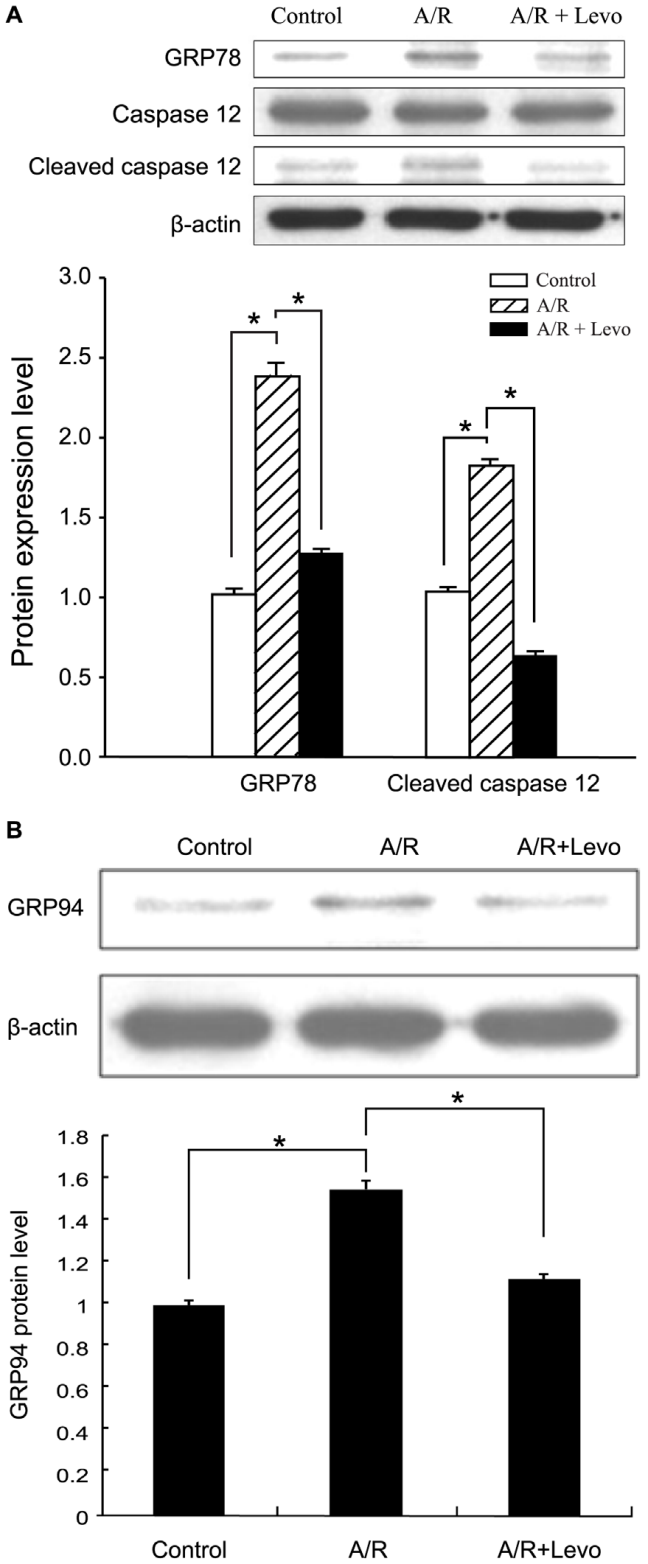
Increased GRP78 expression (6A), GRP94 expression (6B) and caspase 12 activation in anoxia-reoxygenation induced ERS response were abrogated by adding levosimendan, each result obtained from sample of 3×10^6^ cells. Each point represents mean ± S.D. of three independent experiments. *p<0.05 and **p<0.01 versus untreated control and A/R+Levo. A/R: anoxia-reoxygenation; Levo: Levosimendan.

Expression of GRP 78 and GRP94, ERS markers, significantly increased in CPC-derived cardiomyocytes in A/R (Figures 6A, 6B). Caspase 12 cleavage, which activates downstream apoptotic mechanism, also increased during A/R (Figure 6A). Yet levosimendan significantly reduced this GRP 78 and GRP94 expression and caspase 12 activation in A/R (Figures 6A, 6B).

### NCX Knockdown by siRNA Achieved the Same Effect of ERS Response Mitigation during A/R as Levosimendan Administration

**Figure 7:**
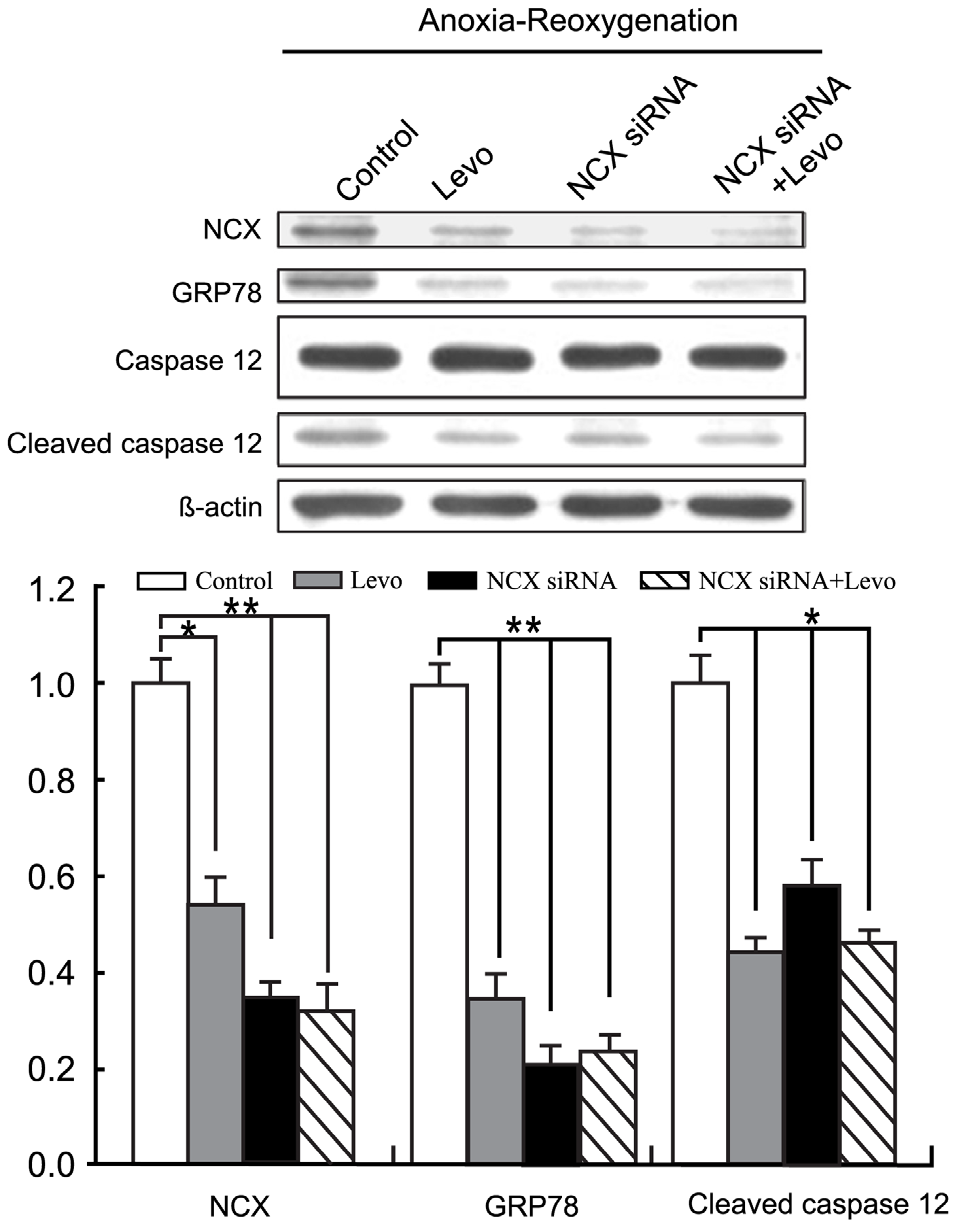
NCX knockdown with NCX siRNA attained effects similar to levosimendan administration in mitigating anoxia/reoxygenation-induced ERS response. Adding levosimendan did not enhance effects obtained by NCX siRNA alone. Results were obtained from samples of 3×10^6^ cells each. Each point represents mean ± S.D. of three independent trials. *p<0.05 and **p<0.01 versus untreated control. Levo: Levosimendan.

To test for increased NCX activity, with attendant increase in [Ca^2+^]_i_ during A/R, as responsible for ERS response in A/R, we performed NCX knockdown experiment. NCX siRNA transfection alone could negate higher GRP 78 expression and caspase 12 activation in A/R (Figure 7), similar to levosimedan addition at reoxygenation (Figure 6). No further decrease in GRP78 expression or caspase 12 cleavage appeared with levosimendan administered in addition to NCX knockdown by siRNA (Figure 7).

## Discussion

As stated, myocardial I/R occurs frequently. Myocardial ischemia may be unpreventable (e.g., acute myocardial infarction), and attendant deleterious effects can only be reduced by keeping the duration of ischemia as short as possible. The start of reperfusion is more or less under the control of clinicians, but there is no effective countermeasure against reperfusion injury. Reperfusion induced cardiomyocyte death has become a significant limiting factor to the achievable result in treating patients with myocardial infarction [3]. Preserving viability of cardiomyocytes has also arisen as a key principle in managing patients with heart failure [11]. In short, there is an unmet demand for cardioprotection in care of cardiac patients; an applicable method to preserve cardiomyocytes is vigorously sought after. In addition, administration of an inotrope is frequently needed to treat manifestations of heart failure in setting of myocardial I/R. Levosimendan has been introduced into clinical usage as an unique Ca^2+^-sentitizing inotrope, and reported to be protective against reperfusion injury in laboratory studies with animal cells. It raises cardiac contractility by increasing sensitivity of troponin C for Ca^2+^ but not [Ca^2+^]i or oxygen consumption [8]. The activation of mitochondrial ATP-sensitive potassium channels by levosimendan might produce pre- or post-conditioning effects and prevent cardiomyocyte necrosis and apoptosis in I/R [24], [25]. It is also a vasodilator, enhancing coronary blood flow while reducing cardiac preload and afterload. These effects benefit myocardium recovering from I/R insult. Using an anti-ischemic inotrope thus remains a possible advantage over conventional β-adrenergic agonists in patients with myocardial I/R. Mechanisms of this levosimendan-conferred cardioprotection remain unclear. Given detrimental effects of intracellular Ca^2+^ overload in I/R, we set out to ascertain how levosimendan affects this pathophysiology. We aimed to determine effects of levosimedan on A/R in human cells, providing stronger and more direct evidence of its protective actions against reperfusion injury in clinical application. With no human cardiomyocyte cell line available, we obtained atrial tissue specimen from patients undergoing cardiac surgery, and followed reported methods to cultivate, propagate, and differentiate progenitor cells into cardiomyocytes for further tests. Such harvesting of endogenous cardiac stem cell from surgical waste has been utilized in a clinical trial recently [26].

Several groups have isolated cardiac stem cells relying on expression of certain markers (c-kit, Sca-1, or Isl1) or particular biological behavior of cells (Hoechst dye efflux or formation of cardiosphere) [27]. Little is known about the interrelationship between stem cell types isolated by diverse means, even less about their numbers and activities in various pathological conditions. It has been reported that number of stem cells rises significantly in hypertrophic myocardium [27]. All our patients show congenital heart malformations and right heart overload; we cannot comment on whether cells acquired from hearts with other diseases would behave differently.

NCX, working in reverse mode, is the main channel for Ca^2+^ influx during I/R. Inhibition of reverse mode NCX activity, with reduced cellular Ca^2+^ accumulation, proves cardioprotective and reduces infarct size [5]. Our results indicated NCX activity (reverse mode) initially reduced after hypoxic insult to CPC-derived cardiomyocytes, consistent with our previous report [4], and after reoxygenation NCX activity rebounded to above control levels. This should contribute significantly to aggravated intracellular Ca^2+^ overload during the initial phase of reoxygenation and ensuing changes causing cell death. Addition of levosimendan during reoxygenation period abolished this heightened NCX activity and reduced [Ca^2+^]i accumulation, ERS response, and cellular apoptosis. Translocation of NCX away from plasma membrane of CPC-derived cardiomyocytes with leveosimendan present may partly explain decreased NCX activity. The mechanism of levosimendan-induced NCX translocation is not clear and merits further investigation.

Intracellular osmolarity rises in anoxic period due to accumulated anaerobic metabolic product and [Na^+^]i, with ensuant water influx and cell swelling. Reoxygenation aggravates cellular swelling due to rapid normalization (decrease) of extracellular osmolarity and pH (which intensifies intracellular Na^+^ overload through the action of Na^+^/H^+^ exchanger). Cells after anoxia-reoxygenation appeared larger than controls in our experiments. Lighter-stained nuclei might result from dilutional effect, as these cells were swollen. Levosimendan will contribute to [Na+]i accrual in reoxygenation, as it inhibits outward Na+ transport via reverse mode NCX activity.

Intense ERS obviously emanates from A/R injury, with its attendant deprivation of substrate and energy source and perturbation of protein synthesis process. Greater expression of GRP78, GRP94 and activation of caspase 12 in results clearly indicated this reaction. Surprisingly, levosimendan could return GRP78, GRP94 expression and caspase 12 activation to control levels, a mitigation of ERS response. NCX knockdown by siRNA could achieve a similar effect. Adding levosimendan to NCX knockdown yielded no synergistic effects. This indicated increased [Ca^2+^]i in cellular A/R plays a crucial role in occurrence of ERS, and NCX activity was responsible for this increase in [Ca^2+^]i. Concomitant to this ERS response alleviation, there was also reduced cardiomycyte apoptosis, as ascertained by FACS analysis. In short, results suggest levosimendan inhibiting reverse-mode NCX activity to protect CPC-derived cardiomyocytes from A/R-induced ER stress and cell death. Further studies must confirm this result to enhance understanding of I/R injury mechanisms.

## References

[1] Verma S, Fedak PW, Weisel RD, Butany J, Rao V, et al (2002) Fundamentals of reperfusion injury for the clinical cardiologist. Circulation 105(20): 2332–2336. 12021216 10.1161/01.cir.0000016602.96363.36

[2] Bolli R, Becker L, Gross G, Mentzer R Jr, Balshaw D, et al (2004) NHLBI Working Group on the Translation of Therapies for Protecting the Heart from Ischemia. Myocardial protection at a crossroads: the need for translation into clinical therapy. Circ Res 95: 125–134. 15271864 10.1161/01.RES.0000137171.97172.d7

[3] Yellon DM, Hausenloy DJ (2007) Myocardial reperfusion injury. New Eng1 J Med 357: 1121–1135. 10.1056/NEJMra071667 17855673

[4] Yang YC, Fann MJ, Chang WH, Tai LH, Jiang JH, et al (2010) Regulation of Sodium-Calcium Exchanger Activity by Creatine Kinase Energy-compromised Conditions. J Biol Chem 285: 28275–28285. 20576602 10.1074/jbc.M110.141424 PMC2934692

[5] Imahashi K, Pott C, Goldhaber JI, Steenbergen C, Philipson KD, et al (2005) Cardiac-Specific ablation of the Na^+^-Ca^2+^ exchanger confers protection against ischemia/reperfusion injury. Circ Res 97: 916–921. 16179590 10.1161/01.RES.0000187456.06162.cb

[6] Kusuoka H, Camilion de Hurtado M C, Marban E (1993) Role of sodium/calcium exchange in the mechanism of myocardial stunning: Protective effect of reperfusion with high sodium solution. J Am Coll Cardiol 21: 240–248. 8417067 10.1016/0735-1097(93)90743-k

[7] Mattiello JA, Margulies KB, Jeevanandam V, Houser SR (1998) Contribution of reverse-mode sodium-calcium exchange to contractions in failing human left ventricular myocytes. Cardiovasc Res 37: 424–431. 9614497 10.1016/s0008-6363(97)00271-x

[8] Papp Z, Edes I, Fruhwald S, De Hert SG, Salmenperä M, et al (2012) Levosimendan: Molecular mechanisms and clinical implications: Consensus of experts on the mechanisms of action of levosimendan. Int J Cardiol 159: 82–87. 21784540 10.1016/j.ijcard.2011.07.022

[9] Shintani-Ishida K, Nakajima M, Uemura K, Yoshida K (2006) Ischemic preconditioning protects cardiomyocytes against ischemic injury by inducing GRP78. Biochem Biophys Res Commun 345(4): 1600–1605. 16735028 10.1016/j.bbrc.2006.05.077

[10] Maytin M, Colucci WS (2005) Cardioprotection: A new paradigm in the management of acute heart failure syndromes. Am J Cardiol 96: 26G–31G. 10.1016/j.amjcard.2005.07.018 16181820

[11] Grossini E, Caimmi PP, Platini F, Molinari C, Uberti F, et al (2010) Modulation of programmed forms of cell death by intracoronary levosimendan during regional myocardial ischemia in anesthetized pigs. Cardiovasc Drugs Ther 24: 5–15. 20162343 10.1007/s10557-010-6217-0

[12] Kersten JR, Montgomery MW, Pagel Ps, Warltier DC (2000) Levosimendan, a new positive inotropic drug, decreases myocardial infarct size via activation of K(ATP) channels. Anesth Analg 90: 5–11. 10624967 10.1097/00000539-200001000-00003

[13] du Toit E, Hofmann D, McCarthy J, Pineda C (2001) Effect of levosimendan on myocardial contractility, coronary and peripheral blood flow, and arrhythmias during coronary artery ligation and reperfusion in in vivo pig model. Heart 86: 81–87. 11410569 10.1136/heart.86.1.81 PMC1729816

[14] Eriksson O, Pollesello P, Haikala H (2004) Effect of levosimendan on balance between ATP production and consumption in isolated perfused guinea-pig heart before ischemia or after reperfusion. J Cardiovasc Pharmacol 44: 316–321. 15475828 10.1097/01.fjc.0000137163.22359.17

[15] Busk M, Maeng M, Kristensen J, Berg JS, Mortensen UM, et al (2006) Effects of levosimendan on myocardial infract size and hemodynamics in a closed-chest porcine ischemia-reperfusion model. Cardiovasc Drugs Ther 20: 335–342. 17122904 10.1007/s10557-006-0294-0

[16] Lilleberg J, Nieminen MS, Akkila J, Heikkila L, Kuitunen A, et al (1998) Effects of a new calcium sensitizer, levosimendan, on haemodynamics, coronary blood flow and myocardial substrate utilization early after coronary artery bypass grafting. Eur Heart J 19: 660–668. 9597417 10.1053/euhj.1997.0806

[17] Scheiermann P, Beiras-Fernandez A, Mutlak H, Weis F (2011) The protective effects of levosimendan on ischemia/reperfusion injury and apoptosis. Cardiovascular Drug Discovery 6: 20–26. 10.2174/157489011794578482 21208156

[18] Smits AM, Vliet PV, Metz CH, Korfage T, Sluijter JPG, et al (2009) Human cardiomyocyte progenitor cells differentiate into functional mature cardiomyocytes :an in vitro model for studying human cardiac physiology Nat Protoc. 4: 232–243. 10.1038/nprot.2008.229 19197267

[19] Wang HH, Li PC, Huang HJ, Lee TY, Lin CY (2011) Peritoneal dialysate effluent during peritonitis induces human cardiomyocyte apoptosis by regulating the expression of GATA-4 and Bcl-2 families. J Cell Physiol 226: 94–102. 20625998 10.1002/jcp.22309

[20] Chang KC, Barth AS, Sasano T, Kizana E, Kashiwakura Y, et al (2008) CAPON modulates cardiac repolarization via neuronal nitric oxide synthase signaling in the heart. Proc Natl Acad Sci USA 105: 4477–4482. 18337493 10.1073/pnas.0709118105 PMC2393814

[21] Nicoletti I, Migliorati G, Pagliacci MC, Grignani F, Riccardi C (1991) A rapid and simple method for measuring thymocyte apoptosis by propidium iodide staining and flow cytometry. J Immunol Methods 139: 271–279. 1710634 10.1016/0022-1759(91)90198-o

[22] Korchak H, Weissmann G, Rutherford L, Rich A, Wilkenfeld C (1982) A carbocyanine dye, DiOC6(3), acts as a mitochondrial probe in human neutrophils. Biochem Biophys Res Commun 108: 1495–1501. 7181903 10.1016/s0006-291x(82)80076-4

[23] Hu D, Kipps T (1999) Reduction in mitochondrial membrane potential is an early event in Fas-independent CTL-mediated apoptosis. Cell Immunol 195: 43–52. 10433796 10.1006/cimm.1999.1513

[24] Garcia-Gonzalez MJ, Dominguez-Rodriguez A, Abreu-Gonzalez P (2007) New pharmacologic options in the treatment of acute coronary syndromes and myocardial ischemia-reperfusion injury: Potential role of levosimendan. Minerva Cardioangiol 55: 625–635. 17912166

[25] Yamada M (2010) Mitochondrial ATP-sensitive K+ channels, protectors of the heart. J Physiol 588 (2) 283–286. 20080515 10.1113/jphysiol.2009.179028 PMC2821723

[26] Bolli R, Chugh AR, D’Amario D, Loughran JH, Stoddard MF, et al (2011) Cardiac stem cells in patients with ischaemic cardiomyopathy (SCIPIO): initial results of a randomised phase 1 trial. Lancet. 378: 1847–1857. 10.1016/S0140-6736(11)61590-0 PMC3614010 22088800

[27] Barile L, Messina E, Giacomello A, Marbán E (2007) Endogenous cardiac stem cells. Prog Cardiovasc Dis. 50: 31–48. 10.1016/j.pcad.2007.03.005 17631436

